# #NoDaysOff: examining the relationship between exercise habits and lifestyle-based consumer behavior

**DOI:** 10.3389/fpsyg.2025.1639930

**Published:** 2025-08-29

**Authors:** Minjeong Kim

**Affiliations:** Division of Business, College of Business, Chosun University, Gwangju, Republic of Korea

**Keywords:** consumer lifestyle, exercise, social media engagement, value-driven consumption, experience goods, search goods

## Abstract

This study explores how contemporary lifestyle characteristics shape consumption behaviors. Specifically, it focuses on three key traits: consistent exercise habits reflecting a health-conscious lifestyle, the habitual sharing of daily experiences on social media, and value-driven consumption behaviors aligned with environmental sustainability and fair trade. By examining the main effects, two-way interactions, and a three-way interaction among these variables, the study aims to understand how such lifestyle patterns influence purchase intentions for general consumer goods. A survey of 223 participants was conducted, and regression analysis was used to test the hypotheses. The results reveal that social media engagement significantly predicts purchase intention, and this relationship is moderated by exercise habits—indicating that health-conscious consumers who engage in regular exercise are more influenced by social media. While value-driven consumption did not have a direct effect on purchase intention, it indirectly enhanced purchase intention through its positive interaction with exercise habits. Additional analyses conducted separately for experience goods and search goods showed consistent patterns for search goods; however, the interaction effects were not significant for experience goods. These findings offer insights into the lifestyle-oriented consumer behaviors, emphasizing the role of exercise habits in shaping consumers’ responsiveness to social and value-based influences in the marketplace.

## 1 Introduction

Reflecting broader societal trends, exercise habits have become an essential element for contemporary consumer behavior. Regular exercise is no longer just a health necessity; it is a lifestyle choice that shapes identity, social interactions, and purchasing decisions. The positive effects of exercise are undeniable, highlighting its significant impact on life and the necessity of regular physical activity. As awareness of fitness grows, individuals actively seek effective exercise methods, with the goal of maintaining their health and enhancing their quality of life. In the past, the decline in muscle mass with age was considered a natural part of aging. However, in modern times, this reduction in muscle mass is recognized as a medical illness (Marques et al., 2024; Peng et al., 2024). Consequently, consumers are learning effective exercise methods to prevent muscle loss as they age and are striving to maintain better health (Shen et al., 2023).

Particularly after COVID-19, the emphasis on health and concerns about the quality of life have increased, leading to the growth of the exercise-related market. For example, the number of consumers actively investing in health and fitness has been rising, and this phenomenon is referred to as the “Dumbbell Economy” (Song, 2020). The term “Dumbbell Economy” describes the trend where high interest in health and exercise drives a strong desire for a healthy lifestyle, leading to the growth of gyms, Pilates studios, fitness equipment, and health-related technologies and products (Park, 2022). Additionally, exercise habits contribute significantly to the formation of a substantial market, influencing purchasing decisions and consumption patterns.

Despite this trend, previous research has primarily focused on the direct effects of exercise habits on purchasing behavior related to health products habits (Cho and Chiu, 2021; Chiu and Choi, 2018; Lee et al., 2011a, 2011b). By increasing fitness-conscious consumption, prior studies have overlooked the moderating role of exercise habits in general product categories. Exercise habits themselves can serve as a deeper psychological indicator—reflecting an individual’s core values, discipline, and behavioral tendencies. Therefore, this study aims to explore whether exercise habits influence purchase decisions beyond fitness-related products, extending to general consumer goods. This study focuses on consumer behavior patterns that are shaped by individuals’ recurring habits and lifestyle characteristics—such as regular exercise, value-driven purchasing, and social media engagement. These patterns reflect a lifestyle-based segmentation approach that has been widely adopted in consumer research (Plummer, 1974). To examine this, I investigate how exercise habits interact with value-driven consumption tendencies and social media engagement behavior to shape purchase intention.

Exercise habits may be shaped by consumers’ characteristics like intrinsic values and beliefs. For example, the growing practice of sharing exercise routines online—through hashtags like *#NoDaysOff*’—demonstrates how fitness behaviors are integrated into social identity and digital engagement. In addition, exercise habits, value-driven consumption, and social media engagement represent three major variables with complex interactions. Thus, this research investigated the interactions among these three variables. These three variables-exercise habits, value-driven consumption, and social media engagement-are not independent characteristics, but interrelated components of modern lifestyle behavior. Prior studies suggest that individuals who engage in regular physical activity tend to have higher levels of self-regulation and health consciousness, which are often associated with ethical and sustainable consumption (Plummer, 1974). Moreover, both health-related and value-driven behaviors are commonly expressed and reinforced through social media platforms, where users share their lifestyles and consumption choices as a form of self-presentation and social signaling (Smith and Sanderson, 2015).

Moreover, rather than focusing solely on health-related products, this study explores how exercise habits influence preferences for general consumer goods, such as experience goods (e.g., hotel accommodations) and search goods (e.g., wireless TV electronics). This perspective enables a more comprehensive understanding of how lifestyle factors shape broader purchasing patterns. Exercise habits also could be regarded as a significant characteristic of consumers, serving as a critical indicator of their overall consumption traits. Furthermore, additional analyses were conducted to examine how value-driven consumption, social media engagement, and exercise habits influence purchase intentions for each product category.

This research contributes by being the first to examine the impact of exercise habits, which require significant time and effort, on consumers’ purchase intentions for general products. This study highlights how exercise habits can serve as a critical cue of consumer lifestyle in understanding purchase decision-making. Additionally, the study emphasizes the importance of considering the interaction between exercise habits and other factors such as consumers’ value-driven consumption beliefs and social media engagement. By considering such interactions, we can contribute to a better understanding of consumer purchasing behavior. Furthermore, by demonstrating different influences across product categories, this study suggests that purchase decisions may vary depending on the type of product. In conclusion, this research underscores the role of exercise habits as a key indicator of consumer behavior, illustrating how they influence and relate to purchase decisions for both health-related and general products.

## 2 Research background and hypotheses

### 2.1 Exercise habit

Exercise habits are not merely health-related behaviors; they represent a fundamental aspect of consumer identity, influencing a wide array of purchase decision. This characteristic can be understood as a type of consumer lifestyle, and this lifestyle segment consumers can be observed as common behavioral traits. Consumers who engage in regular exercise exhibit patterns of discipline, goal-setting, and consistency—traits that extend beyond fitness and into their broader consumption habits. Just as exercise requires commitment and routine, consumers with strong exercise habits are more likely to seek products and services that align with their structured, goal-driven lifestyles.

First, these consumers demonstrate a strong interest in maintaining healthy eating habits and engage in regular physical activity (Cobb-Clark et al., 2014; Bloch, 1984). Consumers with exercise habits tend to be interested in products related to dietary management and health monitoring technologies (Divine and Lepisto, 2005). Based on their values and beliefs, they actively seek information on what foods to consume and effective stress management techniques (Ali and Ali, 2020). Similarly, they represent interest in what types of exercise methods or equipment can improve their performance and stay informed about related products (Divine and Lepisto, 2005).

Furthermore, consumers with well-established exercise habits tend to exhibit a higher internal locus of control (Cobb-Clark et al., 2014). Locus of control refers to the belief that one’s mindset and actions significantly influence behaviors and outcomes (Rotter, 1966). These individuals place greater importance on internal locus of control, perceiving their own actions as the most critical determinant of outcomes rather than external influences (Cobb-Clark et al., 2014). Therefore, consumers with well-formed exercise habits are more likely to demonstrate higher levels of independence in their actions, invest significant effort, and take responsibility for their results. Additionally, these consumers have been found to adhere more effectively to medication intake and health-related behavioral indicators that contribute to health promotion (Stanton, 1987). Hence, these consumers are capable of voluntarily and consistently engage in exercise, making them a group of consumers with a high level of control.

Similarly, well-established exercise habit consumers show a tendency toward an instrumental attitude. Instrumental attitude, derived from the Theory of Planned Behavior (TPB), refers to the evaluation of how effective and useful one’s behavior is in achieving specific goals and outcomes (Hagger and Hamilton, 2021; Ajzen, 1991). Engaging in exercise, for example, is perceived as an effective and practical behavior for promoting one’s future health (de Bruijn, 2011). As a result, individuals who exercise regularly view it as a planned behavior that leads to positive outcomes, encouraging them to sustain a consistent workout routine. In other words, from an intrinsic motivational perspective, exercise strongly reflects an instrumental attitude, which highlights the practical and rational judgment of behaviors (James and Ross, 2004; Weiner, 1986). This characteristic is a defining trait of consumers who prioritize the utility and effectiveness of their actions.

Finally, consumers who engage in consistent exercise tend to place greater interest and value on the enjoyment derived from social interactions and processes. For these individuals, exercise is not merely an activity; it serves as a critical factor in fulfilling their emotional and social satisfaction (Yan et al., 2023). Exercise reinforces intrinsic motivation by fostering social support, which, in turn, sustains long-term engagement (Yan et al., 2023). Furthermore, exercise enhances subjective wellbeing and happiness by strengthening social relationships and increasing satisfaction with life (Liao et al., 2023).

This consistent commitment to exercise reflects shared psychological and behavioral traits that extend beyond fitness and directly influence purchasing behavior. Regular exercise does not function in isolation; rather, it interacts with other consumer attributes such as value-based consumption and social media engagement, collectively shaping product and service preferences. Recognizing exercise as a core consumer characteristic offers deeper insights into purchasing motivations, allowing for a more comprehensive understanding of how structured health-oriented lifestyles drive broader consumption patterns and decision-making processes.

### 2.2 Value-driven consumption

Value-driven consumption refers to the process of evaluating products and services not solely based on their functional attributes but also on their alignment with the consumer’s personal criteria for utility and positivity in shaping future outcomes (Mason et al., 2023). Consumers increasingly consider factors such as sustainability, corporate social responsibility, and ethical production practices as key criteria in their purchasing decisions (Prince et al., 2024). For instance, individuals with strong ethical commitments may actively seek fair-trade products or environmentally friendly goods. By choosing products that are consistent with their values, consumers reinforce their identity and express their personal beliefs through their consumption choices (Girish, 2011).

Moreover, value-driven consumption provides psychological reinforcement by validating a consumer’s choices, leading to greater satisfaction with the overall consumption experience (Wang et al., 2023). This emotional reinforcement strengthens brand loyalty, enhances trust, and fosters positive product evaluations (Zhang et al., 2024; Kumar et al., 2024; Wang et al., 2023). In consequence, companies are increasingly integrating value-based messaging into their marketing strategies. Highlighting aspects such as sustainability and ethical production in advertisements can effectively engage value-driven consumers and enhance their purchase intentions.

Ultimately, value-driven consumption serves as a critical consumer characteristic of lifestyle that significantly influences purchasing decisions. Marketing researchers and practitioners are leveraging these characteristics to design communication strategies that align with consumer values, facilitating engagement and purchase intentions.

### 2.3 Social media engagement

Consumers use social media for various purposes and reasons, particularly to search for and share information. First, consumers actively make use of social media to acquire diverse information related to products, brands, and trends (Zhao and Zhang, 2017; Hamid et al., 2016). By exploring the latest updates and personally relevant information, they can make informed decisions that may lead to future purchases (Kim et al., 2014). For example, consumers are influenced by social influence through the reviews and recommendations of others, which can drive their purchasing decisions.

Social media is also used for entertainment and leisure purposes (Brandtzag and Heim, 2009; Ellison et al., 2007). Consumers may engage with content such as trending memes or videos for enjoyment and share these within their social communities, thereby amplifying their social networks (Lauretta Edwards and Bruce, 2002). For instance, by sharing issues that are popular among their peers or within their social groups, consumers can significantly enhance the reach of such trends (Martocchio and Webster, 1992). Additionally, consumers may be influenced by posts from influencers or other prominent peoples on social media platforms, which they then share or act upon (Luarn and Chiu, 2016).

Lastly, social media serves as a platform for self-expression, allowing consumers to showcase their values, lifestyles, and identities (Brandtzag and Heim, 2009; Ellison et al., 2007). For example, users might post about their travel experiences or share daily updates on completed fitness routines, thereby reinforcing their personal image. This form of self-expression provides satisfaction through shared information and helps build a distinct and credible identity within the social media platform (Zhao and Zhang, 2017).

These social media engagement behaviors are closely linked to consumer purchasing behaviors. As illustrated, social media enables the exchange and dissemination of information that actively influences purchasing decisions (McKnight et al., 2002). For instance, consumers may be motivated to purchase specific products based on influencer recommendations or learn exercise techniques from fitness-related content shared by others. Additionally, through social media, individuals may share products owned by their reference groups or products aligned with their identities, which in turn can encourage others to make similar purchases (Zhao and Zhang, 2017). Therefore, social media engagement behaviors should be regarded as an integral characteristic of consumers, significantly influencing their purchasing decisions and overall consumer behavior.

### 2.4 Research hypotheses

Based on the previous literature, this study establishes the following research hypothesis. First, the influence of consumers’ value-driven consumption tendencies on purchase intention can be significantly amplified through interactions with exercise habits. Value-driven consumption refers to consumers’ propensity to select products and services that resonate with their ethical values and self-identity (Prince et al., 2024; Mason et al., 2023). Consumers exhibiting these tendencies tend to exercise deliberate control over their purchasing decisions, driven by intrinsic motivations, and consequently demonstrate a strong inclination toward products that align with their value system.

In this context, regular exercise acts as a behavioral catalyst that enhances consumers’ psychological wellbeing and emotional state, ultimately influencing their consumption patterns. Consumers who engage in regular exercise are likely to experience an improved sense of self-control, psychological satisfaction, and self-efficacy (James and Ross, 2004). These benefits not only empower them to make more deliberate and consistent decisions but also bolster their confidence in choosing products that reflect their core values. Thus, exercise habits function as an environmental moderator, interacting with value-driven consumption tendencies to further strengthen their effect on purchase intention.

Furthermore, regular exercise reinforces feelings of autonomy and achievement, thereby encouraging consumers to align their behaviors more closely with their personal values. This alignment intensifies their commitment to selecting products that mirror their ethical beliefs and identity, resulting in a synergistic enhancement of purchase intention. Consequently, the interplay between exercise habits and value-driven consumption tendencies is posited to produce a compounded positive effect on consumers’ purchase intentions.

*H1*: Exercise habits strengthen the relationship between consumer value-driven consumption and purchase intention.

Consumers who use social media tend to explore information about products and services through their chosen platforms, processing content such as positive reviews or influencer recommendations based on perceived credibility (Ellison et al., 2007). This process builds trust in products or brands, leading to a positive attitude and increasing the likelihood of purchase intention. In particular, consumers gain psychological assurance regarding their product choices through the social proof provided by social media.

At this point, exercise habits as a behavioral factor can play a pivotal role in moderating the relationship between social media engagement and purchase intention. Regular physical activity boosts self-efficacy by instilling a sense of accomplishment and reinforcing the belief that one can achieve goals and maintain control over one’s actions (Lee, 2024). Furthermore, the psychological satisfaction derived from exercise enables consumers to process social media content more positively, thereby strengthening their motivation to translate this information into purchasing behavior (Lee, 2024).

Moreover, exercise habits may promote self-expression behaviors on social media. For example, when consumers share their “No Days Off” posts, they often receive likes, comments, or praise from other users, fostering a high level of social support (Lee, 2024). This interaction not only provides psychological satisfaction and rewards but also reinforces their engagement on the social media platform (Brandtzag and Heim, 2009). In conclusion, the interaction between social media engagement behaviors and exercise habits creates a synergistic effect, thereby strengthening consumers’ purchase intentions. This dynamic illustrates that social media usage extends beyond mere information exploration, as behavioral traits and psychological satisfaction combine to serve as powerful drivers of consumer purchasing behavior.

*H2*: Exercise habits strengthen the relationship between social media engagement and purchase intention.

Building on the preceding hypotheses, the present study adopts a stepwise conceptual framework to examine the interplay among exercise habits, value-driven consumption, and social media engagement in predicting purchase intention. Although each of these lifestyle-related variables has been individually linked to consumer behavior in prior research, their combined effects, particularly in the context of general consumer goods, have not been sufficiently explored. Hypotheses 1 and 2 are formulated to examine the two-way interaction effects of exercise habits with value-driven consumption (H1) and with social media engagement (H2), respectively. These hypotheses are grounded in the premise that exercise habits reflect a health-oriented self-identity that can enhance the influence of both intrinsic value alignment and digital lifestyle cues on consumer decisions.

Therefore, hypothesis 3 extends this logic by proposing a three-way interaction among the three variables. This hypothesis suggests that the influence of social media engagement on purchase intention is most pronounced when both exercise habits and value-driven consumption beliefs are high—reflecting a convergence of health, values, and social expression as drivers of consumption. This approach assumes that consumer behavior is shaped not by isolated traits but by the dynamic interaction of multiple lifestyle dimensions. Therefore, following established recommendations for interaction modeling (Aiken and West, 1991), the three-way interaction in H3 is not presented in isolation, but as a higher-order extension that builds upon and integrates the lower-order interactions tested in H1 and H2. Accordingly, the interpretation of H3 is meaningful only in the context of the previously established two-way relationships.

In conclusion, this study proposes that the three consumer characteristics—value-driven consumption, social media engagement, and exercise habits—interact in a complex three-way relationship. All three factors share a strong emphasis on independence, intrinsic motivation, and self-control, distinguishing them from external or situational influences. Furthermore, individuals exhibiting these traits often prioritize expressing their identity and maintaining autonomy in their decision-making, suggesting that the combined influence of these factors may significantly impact purchase intentions.

**Figure 1:**
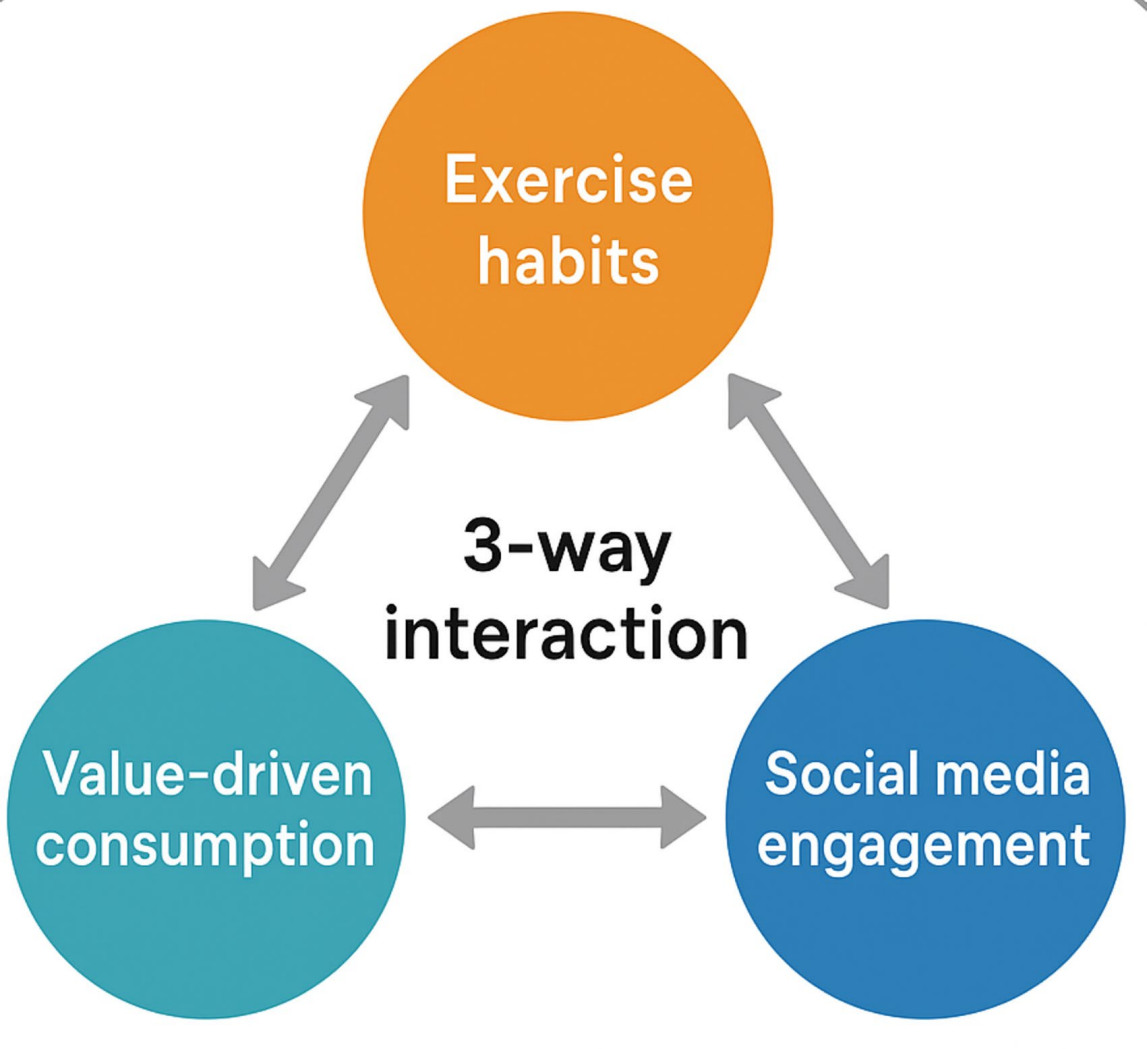
Conceptual model of research.

For example, a consumer who regularly exercises and encounters a product that aligns with their value-driven consumption tendencies is likely to engage more actively with social media to explore reviews and recommendations. This increased engagement is not merely a behavior of information acquisition-it reflects a broader psychological process in which the consumer experiences enhanced self-efficacy, a stronger sense of identity congruence, and a desire for self-expression. Regular exercise fosters a disciplined mindset and intrinsic motivation, which amplify the consumer’s confidence in their value-driven choices. When such consumers also engage in social media behaviors-seeking validation or reinforcing their identity-they are more likely to translate that alignment between product values and personal beliefs into actual purchase intention. Therefore, the interaction of these three variables forms a reinforcing psychological loop, where consistency in lifestyle, personal values, and social validation collectively intensify the purchase intention. And the conceptual model of this study is presented in Figure 1.

*H3*: There is a three-way interaction among exercise habits, value-driven consumption beliefs, and social media engagement on purchase intention.

## 3 Methodology

### 3.1 Data research design

This study aimed to examine the influence of three consumer characteristics (value-driven consumption, social media engagement, and exercise habits) on product purchase intentions. Unlike previous studies that primarily focused on exercise or health-related products, this research investigated whether these characteristics could also impact the purchase of search and experience goods. Therefore, a survey was conducted to collect data on consumers’ lifestyle characteristics related variables, and purchase intentions.

The survey was collected both online and offline during the final week of September 2024. At the beginning of the survey, participants responded to questions about the key variables: value-driven consumption, social media behavior, and exercise habits. Following this procedure, a scenario was presented a scenario to evaluate purchase intentions for general products. At this point, the products were presented as being manufactured using eco-friendly technology, generating positive social impact, and aligning with current trends. For example of search goods, participants were shown a portable TV product, and for experience goods, they were presented to a promotional hotel stay voucher. Each participant was exposed to only one of the stimuli in a between-subjects design. Participants were given sufficient time to review the stimuli before being asked about their purchase intentions.

Furthermore, the survey targeted general Korean consumers aged 20 and above. Participants were recruited through an online research agency using a panel-based system, and reward points were provided in accordance with the agency’s compensation policy. For offline participants, the survey was administered via their own electronic devices, and a small token of appreciation, such as a coffee coupon, was provided upon completion. Given that the survey was conducted in Korean, the sampling was limited to Korean nationals using a randomly selected sampling procedure.

At the beginning of the survey, respondents were informed of the estimated completion time and were given the option to voluntarily proceed. The questionnaire first assessed participants’ exercise habits, followed by their value-driven consumption tendencies and behaviors related to social media engagement. Participants were then randomly assigned to one of two product conditions in a between-subjects design, both representing general consumer goods. Each scenario highlighted eco-friendly product features—such as ESG-based management and sustainable materials in the case of experience goods (e.g., eco-friendly bedding), and eco-smart production systems and low-carbon distribution channels in the case of search goods. In subsequent, participants’ purchase intentions were measured. Finally, the survey included additional items to assess control variables and collect descriptive demographic information, after which the questionnaire was concluded. Demographic information, such as gender, age, occupation, income, education, marital status, and leisure activity, was collected. Furthermore, participants reported their daily internet usage to account for potential confounding variables and control for the broader effects of respondent characteristics on purchasing decision behavior. In conclusion, a total of 223 responses were collected, and the data were analyzed using cross sectional regression analysis.

To ensure the adequacy of the sample size, an *a priori* power analysis was conducted using G*Power 3.1. Based on a linear multiple regression model with 15 predictors (including main effects, interaction terms, and control variables), a medium effect size (f^2^ = 0.15), a significance level of 0.05, and power of 0.80, the minimum required sample size was calculated to be approximately 103. Given that this study collected data from 223 participants, the sample size is considered sufficient for the intended analyses. The model is represented in Equation 1.

(1) $$\begin{array}{l} {\mathrm{PI}}_{i}=\alpha +\beta_{1}VC_{i}+\beta_{2}SB_{i}+\beta_{3}EH_{i}+\beta_{4}VC_{i}*EH_{i}\\ +\;\beta_{5}SB_{i}*EH_{i}+\beta_{6}VC_{i}*SB_{i}+\beta_{7}VC_{i}*SB_{i}*EH_{i}\\ +\;Control_{i}+\varepsilon_{i} \end{array}$$

$$PI_{i}:Individual\;i^{\prime }s\;purchaseintetnion$$

$$VC_{i}:Individual\;i^{\prime }s\;value-drivenconsumption$$

$${\mathrm{SB}}_{i}:Individual\;i^{\prime }s\;socialmediaengagement$$

$$EH_{i}:Individual\;i^{\prime }s\;exercisehabit$$

Using this model, the direct effects of consumers’ value-driven consumption tendencies (*β*_1_), social media engagement behavior (*β*_2_), and exercise habits (*β*_3_) on purchase intention can be examined. Additionally, by analyzing the interaction effects between value-driven consumption and exercise habits (*β*_4_) as well as between social media engagement behavior and exercise habits (*β*_5_), the moderating effects among consumer characteristics can be examined, enabling the verification of Hypotheses 1 and 2. Also, the three-way interaction term (*β*_7_) among value-driven consumption, social media engagement behavior, and exercise habits will be tested to validate Hypothesis 3. Based on the model, this analysis will provide insights into how consumer characteristics—value-driven consumption tendencies, social media engagement behavior, and exercise habits—collectively influence purchase intention.

### 3.2 Measurement

The questionnaire was originally administered in Korean, and all measurement items were translated from validated scales in previous studies. Instead of directly listing the Korean items, the survey structure is described here based on the conceptual dimensions each set of items was designed to measure. Specifically, items for exercise habits assessed participants’ self-discipline, confidence, and goal orientation toward physical activity. Also, value-driven consumption was measured through items capturing functional, individualistic, and collective value orientations. In addition, social media engagement items covered perceived credibility, interactivity, and behavioral immersion. Finally, purchase intention items assessed willingness, likelihood, and preference toward purchasing the presented product. All items were rated on a 7-point Likert scale.

### 3.3 Exercise habit

Exercise habits were operationally defined as a consumer’s willingness to engage in sustained and regular physical activity, reflecting commitment and active participation (Kim, 2023; Weinberg and Gould, 1955). Based on previous studies, the construct was measured using five items assessing beliefs and confidence in exercise, goal orientation, and the sense of happiness derived from physical activity. The five items used to measure exercise habits included questions assessing whether the respondent had clear exercise goals, perceived self-efficacy associated with exercise, a sense of control over exercise duration and intensity, and whether they engaged in regular and sustained physical activity (Kim, 2023; Weinberg and Gould, 1955). The five-item measure of exercise habits represented a Cronbach’s alpha of 0.82, indicating satisfactory internal consistency.

### 3.4 Value-driven consumption

Value-driven consumption was operationally defined as the tendency of consumers to make purchasing decisions based on their subjective value standards and evaluative beliefs (Gallarza et al., 2011; Vinson et al., 1977). The construct was measured using seven items capturing functional value (e.g., product utility), individualistic value (e.g., autonomy and self-expression), and collective value (e.g., environmental sustainability and social justice) (Holbrook, 2006; Zeithanml, 1988; Jang, 2014; Schwartz, 2012; Lee et al., 2011a, 2011b; Babin et al., 1994). The seven items, developed based on previous studies, assessed various aspects of value-driven consumption. These included preferences for eco-friendly products, the use of reusable shopping bags over disposable plastic bags, favorability toward companies engaged in ESG (Environmental, Social, and Governance) initiatives, trust in fair trade practices, and the degree of preference for products with eco-label certifications (Holbrook, 2006; Zeithanml, 1988; Jang, 2014; Schwartz, 2012; Lee et al., 2011a, 2011b; Babin et al., 1994). The value-driven consumption tendency scale demonstrated a Cronbach’s alpha of 0.81, confirming its credibility, and thus all items were retained for analysis.

### 3.5 Social media engagement

Social media engagement refers to the extent to which consumers continuously interact with social media platforms, characterized by trust in shared information and active participation behaviors (Chen and Dhillon, 2003; Gefen et al., 2003; Gruen et al., 2000; Jarvenpaa et al., 1999). In this study, the construct was assessed through five items encompassing key aspects such as interactivity with content, perceived ease of use, credibility of the platform, and user immersion (Subrahmanyam and Greenfield, 2008; DeWulf et al., 2006; Kim and Stoel, 2003; Blattberg and Deighton, 1991). Social media engagement five items designed to capture participants’ overall attitudes and behaviors related to their use of social media platforms. Specifically, the questions examined the degree of emotional connection or sense of belonging to the platform, the perceived credibility and reliability of the information encountered, the level of enjoyment experienced during use, the extent of perceived interactivity with content and other users, and the perceived usefulness of the platform in everyday life (Kim et al., 2014). The five items measuring social media engagement demonstrated a Cronbach’s alpha of 0.84, indicating that the scale achieved acceptable internal consistency.

### 3.6 Purchase intention

In this study, purchase intention was used a s the dependent variable. The purchase intention items were composed of identical questions to both experience and search goods, formulated with reference to previous studies (Song and Moon, 2023). The operational definition of purchase intention is the degree to which an individual intends to purchase a given product or service (Song and Moon, 2023). Accordingly, the questions were designed to assess the willingness to purchase, the likelihood of purchase, and the extent to which the product satisfies consumer needs and desires. Consequently, a total of four items were finalized for measurement. Purchase intention was measured using four items that assessed participants’ willingness to purchase the product, their desire to own it, and the perceived likelihood of making a purchase (Song and Moon, 2023). The four items of purchase intention demonstrated a high Cronbach’s alpha of 0.92, confirming strong internal consistency.

### 3.7 Control variables

Variables that could potentially influence purchase intention were considered and controlled. Therefore, demographic factors such as gender, marital status, age, occupation, income, and education level were examined, along with hobbies and leisure activities, such as reading books and watching movies. Furthermore, Data on daily internet usage was collected to assess the extent of exposure to products and services on the specific online platform. These details are presented in Table 1 and all these variables are analyzed by dummy variables or ordinal scale.

**Table 1:** Control variables.

Variables	Contents
Gender	Male, Female, Prefer not to respond
Marital status	Married, Unmarried, Declined to answer
Age	Age groups in 10 year intervals
Occupation	Korean Standard Classification of Occupations (KSCO)
Income	Income ranges in increments of 2 million won (₩)
Education	Middle school graduate or below, High school graduate, University or college graduate, Graduate school graduate
Hobbies and leisure activities	Reading, Travel, Listening to music, Watching movies, Other activities
Daily internet usage (per day)	Less than 1 h, 1–3 h, 3–5 h, more than 5 h

## 4 Results and discussion

### 4.1 Results

The results of this study are presented in Table 2. Model 1 represents the baseline model, which includes only control variables. In Model 2, the main variables (exercise habits, value-driven consumption, and social media engagement behavior) were included in the analysis. In Models 3 and 4, when examining the interactions between the main variables, multicollinearity could occur. Therefore, the main variables were mean-centered before conducting the interaction analysis. Consequently, all variance inflation factor (VIF) values were below 2 in Models 3 and 4, confirming that multicollinearity was no longer a concern in the analysis.

**Table 2:** Regression results.

Variables	Model 1	Model 2	Model 3	Model 4 (Full model)
Exercise habit		0.203**	0.306***	0.333***
Value-driven consumption		0.214*	0.049	0.027
Social media engagement		0.332***	0.385***	0.365***
Exercise habit * Value-based consumption			0.158**	0.139*
Exercise habit * Social media engagement			0.187***	0.181***
Value-based consumption * Social media engagement			−0.105	−0.053
3-way interaction				0.067*
(Constant)	3.633**	1.058	4.697***	4.439***
Gender	−0.325	−0.176	−0.108	−0.034
Marital status	0.192	0.156	0.201	0.224
Age	−0.236*	−0.285**	−0.281**	−0.278**
Occupation	0.089	0.088	0.056	0.045
Income	−0.062	−0.101	−0.130	−0.120
Education	0.371	0.237	0.174	0.217
Reading	0.076	0.006	−0.087	−0.096
Travel	0.320	0.015	0.097	0.095
Listening to music	−0.066	−0.145	−159	−0.191
Watching movies	0.386*	0.358*	0.284	0.294
Other activities	0.092	−0.221	−0.171	−0.180
Daily internet usage (per day)	0.349**	0.329**	0.267**	0.269**
Adjusted R^2^	0.135	0.312	0.378	0.388
F	3.902	7.733	8.538	8.431
*N*	223	223	223	223

***p < 0.001, **p < 0.01, *p < 0.05.

To verify the hypotheses, the findings demonstrate a significant positive interaction between exercise habits and value-driven consumption tendency in predicting purchase intention (*β*_4_ = 0.139, *p* = 0.01), supporting Hypothesis 1. This suggests that consumers who are highly value-driven tend to find personal meaning in their purchase decisions, relying on their own beliefs and standards. When these tendencies are paired with consistent exercise habits-often linked to a strong sense of self-efficacy-they seem to reinforce each other and further strengthen purchase intention. However, value-driven consumption on its own did not significantly influence purchase intention. Although the product in the scenario was described as eco-friendly and socially responsible, participants may have seen that information as more of a marketing message than a meaningful product feature. As a result, their responses may have been shaped more by their existing values than by the scenario itself.

Hypothesis 2 was also supported by a significant interaction between social media engagement and exercise habits (*β*_5_ = 0.181, *p* = 0.003). Consumers who actively use social media and maintain exercise routines show enhanced purchase intention, likely due to their motivation to share behaviors, seek validation, and express identity through digital platforms. In addition, hypothesis 3 addressed the joint influence of all three variables. The three-way interaction among exercise habits, value-driven consumption, and social media engagement was statistically significant (*β*_7_ = 0.067, *p* = 0.043). This suggests a reinforcing mechanism: consumers who are value-driven, socially engaged, and exercise-active experience greater alignment between identity and consumption, leading to stronger purchase intentions.

While value-driven consumption tendencies were expected to positively influence purchase intention, the effect was not statistically significant. One plausible explanation is that although the product descriptions emphasized eco-friendly features and ESG-driven business practices, participants may not have perceived these as sufficiently credible or distinctive to activate their value orientations. Also, prior research suggests that value-based responses are more likely when consumers perceive clear personal relevance, emotional resonance, or strong authenticity in the message (Sparks and Shepherd, 1992). In this study, the scenario presentation was relatively neutral and generalized. As a result, it may have lacked the motivational cues necessary to activate value-driven reasoning, leading participants to rely more on habitual or affective responses instead. In consequence, the non-significant effect may reflect a gap between latent value tendencies and actual behavioral intention when message credibility or contextual salience is weak.

Furthermore, this study conducted additional analyses to examine whether the observed interaction effects varied by product type—specifically, between search goods and experience goods. The results showed that in the case of search goods, all interaction effects—including both two-way and three-way interactions—remained statistically significant, thereby reinforcing the robustness of the model across product types. However, for experience goods, the interaction between exercise habits and value-driven consumption was not significant (*β*_4_ = 0.042, *p* > 0.1), and the three-way interaction effect also failed to reach significance (*β*_7_ = −0.027, p > 0.1). These findings suggest that, unlike search goods, consumer decisions related to experience goods may not be strongly influenced by value alignment or habitual tendencies. Notably, the interaction between exercise habits and social media engagement remained significant (*β*_5_ = 0.179, *p* < 0.001), indicating that social connection and sharing behaviors continue to play a critical role in shaping purchase intention in the experience goods context (Table 3).

**Table 3:** Additional regression results.

Variables	Search goods	Experience goods
Exercise habit	0.277	0.390***
Value-driven consumption	0.142	−0.059
Social media engagement	0.506***	0.296***
Exercise habit * Value-driven consumption	0.158*	0.042
Exercise habit * Social media engagement	0.180**	0.179***
Value-driven consumption * Social media engagement	0.051	0.031
3-way interaction	0.116*	−0.027
(Constant)	3.102	5.012***
Gender	0.037	−0.020
Marital status	0.074	0.306
Age	−0.240	−0.332*
Occupation	0.158	−0.039
Income	−0.194	0.091
Education	0.575	−0.038
Reading	−0.666	0.026
Travel	0.116	0.022
Listening to music	0.198	−0.395
Watching movies	0.175	0.355
Other activities	−0.223	−0.173
Daily internet usage (per day)	0.344*	0.298*
Adjusted R^2^	0.403	0.351
F	4.023	4.907
*N*	85	137

***p < 0.001, **p < 0.01, *p < 0.05.

### 4.2 Discussion

These findings confirm that exercise habits function not only as health-related behaviors but also as meaningful lifestyle indicators in the consumer domain. Consumers who regularly engage in exercise tend to show greater self-discipline and a strong orientation toward personal values, which leads them to choose products that reflect their own standards and offer a sense of internal fulfillment. Likewise, recent research suggests that regular exercise is associated with higher self-control and perceived behavioral autonomy, both of which are important predictors of consistent, value-based consumption patterns (Jiang and Zhang, 2025; de Ridder et al., 2011). For instance, Jiang and Zhang (2025) found that frequent physical activity significantly improved mood and life satisfaction, mediated by psychological wellbeing.

The results also indicate that when value-driven consumption motives and social media engagement co-occur in individuals with established exercise habits, a synergistic effect on purchase intention emerges. This finding is supported by recent research suggesting that individuals are more likely to act on internalized values when those values are recognized or reinforced through social interaction and feedback (Peng et al., 2024). Therefore, social media helps people express their values through visible consumption, especially when they are aware of how others see them (Peng et al., 2024). For consumers with high lifestyle consistency, such public affirmation may reinforce value-consistent behavior, thereby strengthening purchase intention.

These mechanisms appear particularly influential in the case of search goods, where consumers can clearly evaluate value-congruent attributes. In contrast, for experience goods, value-based considerations appear to weaken, while the social and emotional appeal of sharing experiences online becomes more salient. This may be because experiential consumption is often driven by immediate gratification, emotional resonance, and opportunities for social sharing, rather than internalized value alignment. As such, consumers may place greater emphasis on how the experience can be displayed or validated in their social networks, rather than on whether it reflects their personal values. This shift represents that in digital environments, experiential consumption often becomes a performance and process, with self-presentation and viewers response playing central roles (Shah et al., 2023). Consequently, the influence of value-driven motives may have been diluted in this context, possibly explaining the non-significant interaction effect observed in the case of experience goods.

This pattern suggests that the psychological mechanism underlying purchase decisions for experience goods may be more aligned with emotional and social drivers than with value congruence. Even individuals who strongly identify with ethical values may place less importance on those beliefs when faced with hedonic or highly sharable consumption contexts. In such situations, social validation and momentary satisfaction may override slower, deliberative reasoning associated with internalized values.

In conclusion, this study contributes to the literature by positioning exercise habits as a lifestyle segmentation criterion related internal psychological drivers (e.g., self-efficacy, autonomy) with social behaviors (e.g., digital sharing), offering a more integrated view of how identity-relevant characteristics influence consumer decisions. By representing how exercise habits amplify the effects of value-driven consumption and social media engagement, this research provides empirical evidence that lifestyle-related characteristics can serve as meaningful indicators for predicting complex patterns of consumer behavior. Such findings underscore the need for marketers and researchers to consider not only what consumers buy, but also how their everyday habits, values, and digital interactions work together to shape their motivations and marketplace choices.

## 5 Conclusion

This study examined how exercise habits, value-driven consumption, and social media engagement interact to influence purchase intention, particularly for general consumer goods. It also explored whether these effects vary between search goods and experience goods. This study demonstrates that exercise habits interact with value-driven consumption and social media engagement to influence purchase intention. The significant three-way interaction highlights exercise habits as a meaningful lifestyle-based consumer characteristic. Notably, the interaction patterns differ across product types, with stronger effects observed in search goods. These insights suggest that exercise habits can be effectively used for consumer segmentation and targeted marketing strategies.

For practitioners, the findings suggest that value-oriented messaging may be particularly effective for consumers with strong exercise habits, especially in the context of search goods. For experience goods, enhancing social media engagement through user-generated content may better resonate with exercise-active consumers. Overall, recognizing and leveraging exercise habits as a consumer trait may offer valuable opportunities for both academic and practical advancements in consumer behavior research.

### 5.1 Limitations and future research directions

Despite its contributions, this study has several limitations. First, as a cross-sectional study, it is limited in establishing clear causal relationships between exercise habits and consumer behavior. Future research should consider longitudinal studies or experimental designs to better elucidate the impact of exercise habits on consumer decision-making processes.

Second, exercise habits were measured using a self-reported Likert scale as a continuous variable based on subjective judgment. However, future studies should explore more granular factors such as exercise intensity, frequency, and type to examine their differentiated impact on consumption behavior. For example, the influence of personal exercises (e.g., yoga, jogging) versus team sports (e.g., basketball, soccer) on consumer tendencies may differ, warranting further investigation.

Third, this study was conducted within the Korean consumer market, potentially limiting its generalizability to different cultural contexts. Future research should compare how the relationship between exercise habits and consumption tendencies varies between Western (individualistic) and Eastern (collectivistic) cultures. While random sampling was applied, the study did not employ a stratified sampling approach, which may limit the demographic representativeness across subgroups. Therefore, the generalizability of the findings to other cultural contexts should be interpreted with caution. Additionally, future studies may consider examining the interaction between exercise habits and other major lifestyle segmentation variables to deepen the understanding of consumer classification models. By addressing these limitations, future research can further solidify exercise habits as a key explanatory factor in consumer behavior.

## Data Availability

The raw data supporting the conclusions of this article will be made available by the authors, without undue reservation.
